# Phylogenetically and Spatially Close Marine Sponges Harbour Divergent Bacterial Communities

**DOI:** 10.1371/journal.pone.0053029

**Published:** 2012-12-27

**Authors:** Cristiane C. P. Hardoim, Ana I. S. Esteves, Francisco R. Pires, Jorge M. S. Gonçalves, Cymon J. Cox, Joana R. Xavier, Rodrigo Costa

**Affiliations:** 1 Microbial Ecology and Evolution Research Group, Centre of Marine Sciences, University of Algarve, Faro, Algarve, Portugal; 2 Centro de Investigação em Biodiversidade e Recursos Genéticos, Laboratório Associado, Pólo dos Açores, Departamento de Biologia da Universidade dos Açores, Ponta Delgada, Açores, Portugal; 3 Fisheries, Biodiversity and Conservation Research Group, Centre of Marine Sciences, University of Algarve, Faro, Algarve, Portugal; 4 Plant Systematics and Bioinformatics, Centre of Marine Sciences, University of Algarve, Faro, Algarve, Portugal; 5 Centre for Advanced Studies of Blanes, Girona, Spain; University of New South Wales, Australia

## Abstract

Recent studies have unravelled the diversity of sponge-associated bacteria that may play essential roles in sponge health and metabolism. Nevertheless, our understanding of this microbiota remains limited to a few host species found in restricted geographical localities, and the extent to which the sponge host determines the composition of its own microbiome remains a matter of debate. We address bacterial abundance and diversity of two temperate marine sponges belonging to the Irciniidae family - *Sarcotragus spinosulus* and *Ircinia variabilis* – in the Northeast Atlantic. Epifluorescence microscopy revealed that *S. spinosulus* hosted significantly more prokaryotic cells than *I. variabilis* and that prokaryotic abundance in both species was about 4 orders of magnitude higher than in seawater. Polymerase chain reaction-denaturing gradient gel electrophoresis (PCR-DGGE) profiles of *S. spinosulus* and *I. variabilis* differed markedly from each other – with higher number of ribotypes observed in *S. spinosulus* – and from those of seawater. Four PCR-DGGE bands, two specific to *S. spinosulus*, one specific to *I. variabilis*, and one present in both sponge species, affiliated with an uncultured sponge-specific phylogenetic cluster in the order *Acidimicrobiales* (*Actinobacteria*). Two PCR-DGGE bands present exclusively in *S. spinosulus* fingerprints affiliated with one sponge-specific phylogenetic cluster in the phylum *Chloroflexi* and with sponge-derived sequences in the order *Chromatiales* (*Gammaproteobacteria*), respectively. One *Alphaproteobacteria* band specific to *S. spinosulus* was placed in an uncultured sponge-specific phylogenetic cluster with a close relationship to the genus *Rhodovulum*. Our results confirm the hypothesized host-specific composition of bacterial communities between phylogenetically and spatially close sponge species in the Irciniidae family, with *S. spinosulus* displaying higher bacterial community diversity and distinctiveness than *I. variabilis*. These findings suggest a pivotal host-driven effect on the shape of the marine sponge microbiome, bearing implications to our current understanding of the distribution of microbial genetic resources in the marine realm.

## Introduction

Marine sponges have been the focus of increasing microbiology research interest mainly because of their symbiotic association with abundant and diverse bacteria and production of biologically active secondary metabolites [1], [2]. For so-called High Microbial Abundance (HMA) sponges, it has been shown that up to 38% of sponge wet weight is composed of bacterial cells [3], and that such bacterial abundance surpasses that of seawater by 2 to 4 orders of magnitude [1], [4]–[6]. It has been suggested that HMA sponges harbour several bacteria involved in the production of secondary metabolites, which might, for example, improve protection against predation of the sponge host [1]. The synthesis of bioactive compounds derived from sponge-microbe associations has already been reported for 26 of the 92 families in the Demospongia [7], the most diversified class of the phylum Porifera. Currently, the use of high-throughput sequencing technology is extending our knowledge of microbial diversity in marine sponges, with more than 25 bacterial phyla detected in sponges by this means [8], [9]. Taken together, these features foreshadow marine sponge holobiomes as valuable reservoirs of microbial genetic and metabolic diversity of potential use in biotechnology.

Despite such remarkable advances, current understanding of symbiont community structure in marine sponges remains restricted to certain regions and host species [1]. This holds true for species within the family Irciniidae (Demospongiae, Dictyoceratida), from which the majority of surveys undertaken so far have been limited to tropical latitudes and to the species *Ircinia felix*, *I.* *strobilina*, and *I. ramosa* [8], [10]–[17]. Electron microscopy analyses unveiled abundant and diverse bacterial morphotypes in *I. felix* [10]–[12], whereas five [15] and seven [17] bacterial phyla were revealed in association with *I. strobilina* by cloning-and-sequencing of 16S rRNAgene fragments. By means of high-throughput sequencing, sixteen phyla and 1199 bacterial operational taxonomic units (OTUs) at 95% sequence similarity were found in association with *I. ramosa* [8], highlighting the complexity of the *Ircinia*-associated “bacteriome”. The detection of *Acidobacteria*, *Alpha*- and *Gammaproteobacteria* in adult, larva and juvenile samples of *I. felix* [11] supports the hypothesis that a portion of this microbiota might be vertically transmitted throughout successive host generations.

Conversely, the microbial ecology of temperate irciniids remains underexplored. Only recently a study first approached the diversity of bacterial communities in Mediterranean *Ircinia* spp. - namely *I. variabilis*, *I. fasciculata* and *I. oros* - revealing eight bacterial phyla across these hosts and species-specific OTUs [18]. Because of their global distribution, encompassing both tropical and temperate species, *Irciniidae* sponges constitute a valuable taxon for the study of the ecology and evolution of symbiotic relationships. In addition, a great variety of cytotoxic compounds has been retrieved from *Irciniidae* species, which indicates they are potentially of high biotechnological importance. [19]–[24]. Furthermore, two studies performed with the temperate *I. muscarum* and *I. variabilis* described the production of cyclic peptides by cultivated bacteria [25], [26], whereas psymberin – which resembled the pederin family of polyketides – was recovered from *Psammocinia* sp. and shown to have a bacterial symbiont origin [19], [27]. In this context, addressing microbial diversity and distribution in widespread and chemically complex marine sponges is not only relevant to the study of symbiosis and co-evolutionary relationships, but also bears importance to our understanding of the extent of marine genetic and metabolic resources.

In light of the recent evidence for divergent bacterial communities across different sponge species or even specimens [9], [28], a feature that has also been observed for other eukaryotes that support complex bacterial consortia [29]–[31], this study uses a stringent experimental design to test the hypothesis of host-specific assemblages of dominant symbionts in marine sponges. To this end, we address bacterial abundance and diversity in the temperate marine sponges *Sarcotragus spinosulus* Schmidt, 1862 and *Ircinia variabilis* Schmidt, 1862 (Demospongiae, Dictyoceratida, Irciniidae), two closely related species that co-exist in spatial proximity at the coast of the Algarve (southern Portugal), a region with a Mediterranean climate located in the Northeast Atlantic. We use the 16S rRNA gene as a phylogenetic marker in polymerase chain reaction – denaturing gradient gel electrophoresis (PCR-DGGE) analyses of the domain *Bacteria*, the phylum *Actinobacteria* and the class *Alphaproteobacteria* in these hosts, thus allowing the inspection of bacterial community structures across different taxonomic ranks with concomitant focus on abundant and biotechnologically relevant sponge-associated microorganisms [7], [32], [33]. Phylogenetic analysis of dominant bacterial populations (i.e. PCR-DGGE bands) consistently and specifically found in association with these species is performed, and their status as “sponge-specific bacterial clusters” [34] is verified. We also determine the degree of dissimilarity between sponge-associated bacterial communities and that of their neighbouring bacterioplankton. To assure accurate identification of the target sponges, we infer host phylogenies based on cytochrome oxidase gene sequence relationships. This is the first study of bacterial community structure and diversity in North Atlantic Irciniidae.

## Results

### Sponge Identification

**Figure 1:**
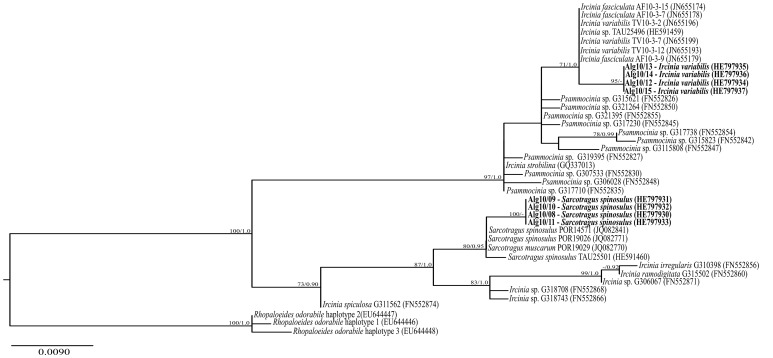
Phylogenetic inference of the Irciniidae family based on the cytochrome oxidase gene, subunit 1. The Maximum Likelihood tree (-ln likelihood: 1383.921591) is shown, with sequences retrieved in this study highlighted in bold. Numbers at tree nodes are bootstrap values and posterior probabilities calculated in Maximum Likelihood and MCMC Bayesian analyses, respectively, and values above 70/0.95 are shown.

Sponge specimens (Fig. S1) were identified as *Sarcotragus spinosulus* and *Ircinia variabilis* based on macro- and microscopic analyses using morphological criteria. Analysis of 636 bp-long sequences of the subunit I of the mitochondrial cytochrome C oxidase (CO1) gene obtained for all specimens (accession numbers HE797930 to HE797937) showed no intraspecific variation among our sequences of *I. variabilis* or *S. spinosulus*, whereas a 4.7% genetic distance (p-distance) was found between our sequences of these two species. Genetic distances between our *S. spinosulus* sequences and those available on NCBI GenBank ranged from 0 to 0.6%, whereas for *I. variabilis* a distance of 0.5% was observed between our sequences and those of *I. variabilis*/*fasciculata* collected in the Northwestern Mediterranean. Phylogenetic reconstructions based both on Maximum Likelihood and Bayesian inference confirmed the identification of our sponge specimens. Indeed, *I. variabilis* and *S. spinosulus* CO1 sequences sampled in this study formed well-supported clades with CO1 sequences from *Ircinia* spp. and *Sarcotragus* spp. retrieved elsewhere (Fig. 1).

### Counting of Heterotrophic Culturable Bacteria

The colony forming units (CFU) counts of heterotrophic bacteria on marine agar revealed no significant difference (*p*>0.05) between sponges species, with 3.21±2.03×10^6^ CFU and 1.63±0.61×10^6^ CFU g^−1^ of fresh sponge for *S. spinosulus* and *I. variabilis,* respectively.

### Epifluorescence Microscopy

**Figure 2:**
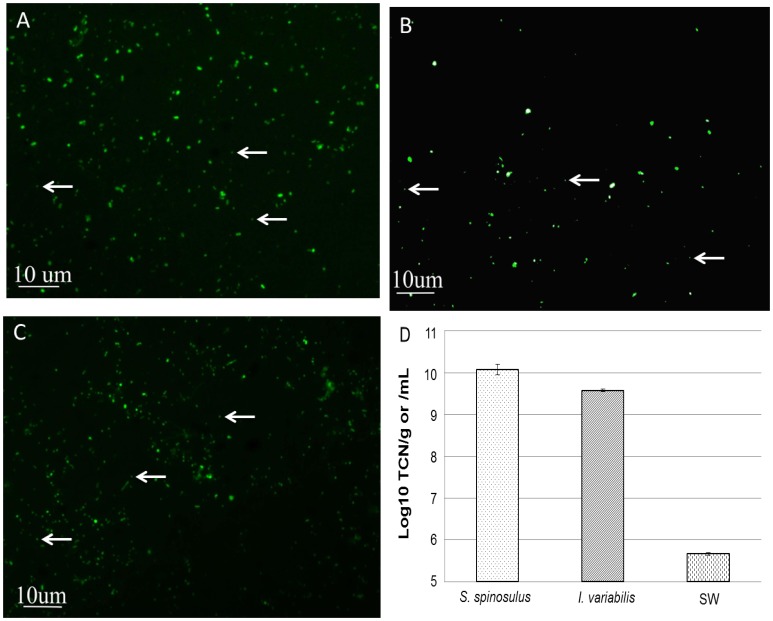
Epifluorescence counts. Microscopy pictures taken from *S. spinosulus* (A), *I. variabilis* (B) and Seawater (C) are shown. Arrows exemplify counted bacterial cells. Values in panel (D) are expressed as means ± standard errors of log-transformed total cell numbers (TCN).

Analyses showed that *S. spinosulus* harboured significantly (*p*<0.05) higher number of prokaryotic cells (average of 1.37×10^10^ cells g^−1^ of fresh sponge), as surveyed in this study, when compared to *I. variabilis* (average of 3.81×10^9^ cells g^−1^ of fresh sponge). The abundance of prokaryotic cells in surrounding seawater (average of 4.63×10^5^ cells mL^−1^) was significantly (*p*<0.05) lower than in both sponge species (Fig. 2).

### PCR-DGGE Analysis of Bacterial Communities

#### (i) Bacteria PCR-DGGE profiles

**Figure 3:**
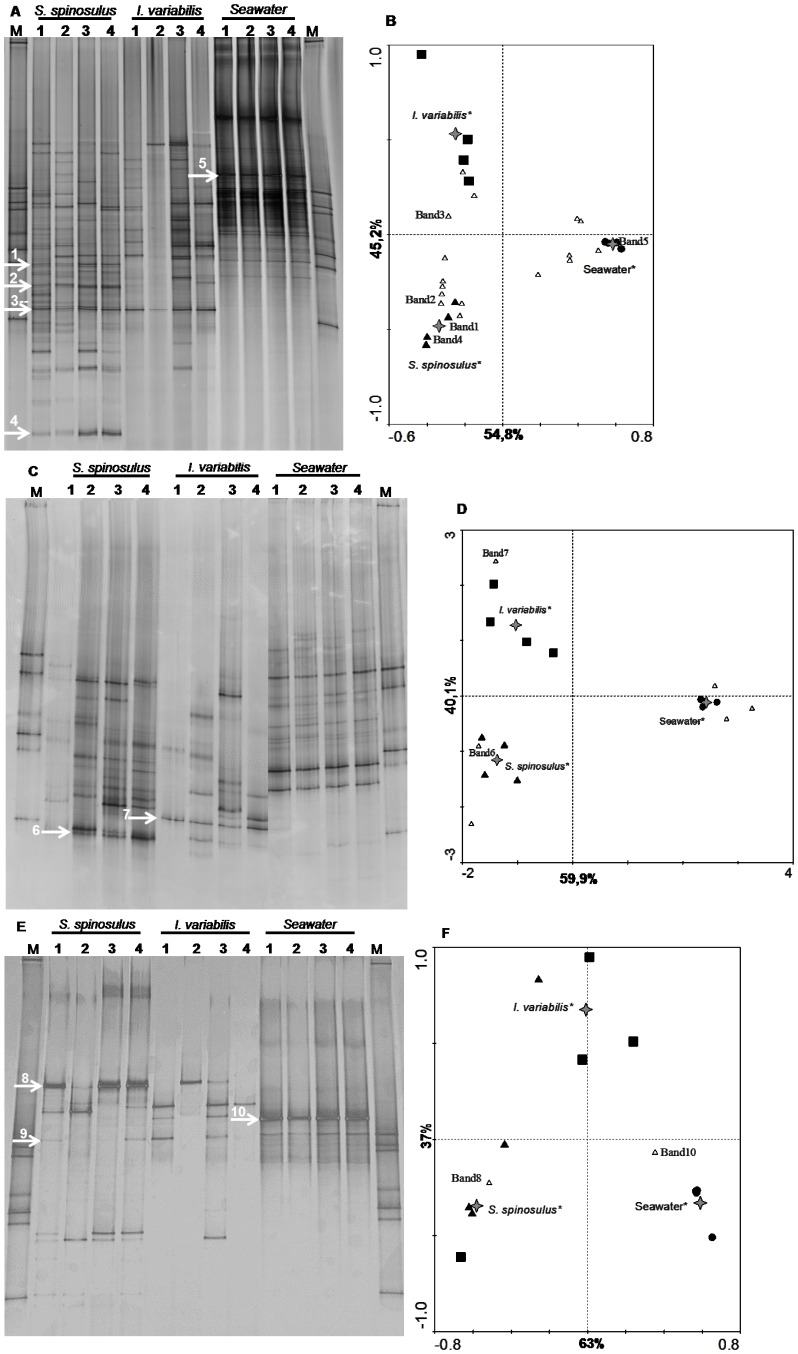
PCR-DGGE fingerprints and canonical analyses. PCR-DGGE 16S rRNA gene fingerprints of *S. spinosulus*, *I. variabilis* and seawater DNA samples generated with “total-community” bacterial primers (A) and specific primer systems for *Actinobacteria* (C) and *Alphaproteobacteria* (E). The arrows indicate bands that were excised from DG-gels and sequenced. Corresponding ordination biplots of PCR-DGGE fingerprints and qualitative environmental variables are shown in panels B, D, F. Symbols: ▴ *S. spinosulus*, ▪ *I. variabilis* and • Seawater. Labels displayed on the diagram axes refer to the percentage variations of PCR-DGGE ribotypes - environment correlation accounted for the respective axis. The “star” symbols represent the centroid positions of the environmental variables in the diagram. Variables that significantly (*p*<0.05) influence the bacterial community composition are indicated by an asterisk.

The bacterial PCR-DGGE profiles of *S. spinosulus* were characterized by 5 dominant bands and a large number of fainter bands (16 to 30) whereas those of *I. variabilis* comprised 1 dominant band in addition to 5 to 26 fainter bands (Fig. 3a, Table S1). Seawater DGGE profiles showed 7 dominant bands and a large number of fainter bands (28 to 30). While the similarity within seawater and *S. spinosulus* replicates was high, profiles of *I. variabilis* specimens displayed large within-replicate heterogeneity (Fig. 3a). Clearly contrasting profiles were observed between seawater and sponge samples, and between both sponge species. Indeed, the UPGMA cluster analysis (Fig. S2a) revealed two main groups, one formed exclusively by all sponge specimens and other containing only seawater samples. These two groups shared less than 10% similarity whereas *S. spinosulus* and *I. variabilis* PCR-DGGE profiles shared around 20% similarity. Ordination via canonical correspondence analysis (CCA) of the DGGE band data and environment variables revealed that sponge species and seawater significantly influenced band variation in the DGGE profiles (*p*<0.05, Fig. 3b). The horizontal axis of the diagram, which accounted for 54.8% of the dependent (i.e. DGGE bands) – independent (i.e. sample classes) variables correlations, mainly distinguished the sponges *S. spinosulus* and *I. variabilis* from seawater (Fig. 3b). The vertical axis grouped replicates from sponge *S. spinosulus* clearly apart from those observed in *I. variabilis*.

#### (ii) Actinobacteria PCR-DGGE profiles

The actinobacterial PCR-DGGE profiles of *S. spinosulus* consisted of few (2 to 4) strong bands along with more than 4 detectable bands, while those of *I. variabilis* comprised 1 to 3 dominant bands along with 1 to 5 fainter bands (Fig. 3c). Significant reduction in actinobacterial diversity and richness were determined for the latter species in comparison with the former (Table S1). A large heterogeneity was observed within *I. variabilis* profiles. In comparison with the sponge fingerprints, the seawater PCR-DGGE profiles displayed higher diversity of bands, especially against *I. variabilis* profiles (Table S1), and contained 3 strong bands along with more than 6 fainter bands, and much lower within-replicate variability (Fig. 3c). Two main groups were revealed by cluster analysis, one formed exclusively by all sponge specimens and other containing only seawater samples (Fig. S2b). These two groups shared about 10% similarity. However, there was also a clear difference between the profiles of both sponge species, which shared *c.* 20% similarity according to cluster analysis. Ordination via CCA discriminated both sponge species and seawater across the horizontal axis of the diagram, which represented around 60% of the overall PCR-DGGE – sample correlations (Fig. 3d). All independent variables (i.e. the sample classes seawater, *S. spinosulus* and *I. variabilis*) significantly (*p*<0.05) affected the PCR-DGGE banding patterns (Fig. 3d).

#### (iii) Alphaproteobacteria PCR-DGGE profiles

The *Alphaproteobacteria* profiles of *S.* *spinosulus* contained 1 to 3 dominant bands, in addition to more than 7 detectable fainter bands, whereas the profiles of *I. variabilis* revealed 1 to 3 strong and fainter bands (Fig. 3e). Significantly greater richness, diversity and evenness were found for *S. spinosulus* alphaproteobacterial PCR-DGGE profiles in comparison with those of *I. variabilis* (Table S1). The seawater profiles showed 2 strong bands along with more than 6 fainter bands. The variability within sponge specimens and among sponge species was relatively high. Conversely, seawater samples displayed highly homogeneous profiles (Fig. 3e). Cluster analysis revealed a clear separation between seawater and sponge samples and high similarity scores for the former (Fig. S2c). The latter grouped into two further clusters in which the visible, higher degrees of within-sample variability could be numerically depicted (Fig. S2c). Sample outliers were detected, as one replicate from *I. variabilis* grouped with a cluster dominated by three *S. spinosulus* samples, and the same effect was observed for one replicate from *S. spinosulus* which clustered with *I. variabilis* specimens (Fig. 3f, S1c). Nevertheless, CCA showed that all factors significantly (*p*<0.05) influenced the patterns of band distribution in alphaproteobacterial PCR-DGGE profiles. Ordination via CCA revealed that 63% of total PCR-DGGE band – independent variables correlations was explained by the horizontal axis of the diagram, which mainly differentiated *S. spinosulus* from seawater (Fig. 3f), whereas the residual variability in the vertical axis of the diagram (37%) discriminated most *I. variabilis* from seawater and *S. spinosulus* samples (Fig. 3f).

### Analysis of Sequences of Dominant and Discriminating PCR-DGGE Bands

#### (i) Bacteria PCR-DGGE bands

**Figure 4:**
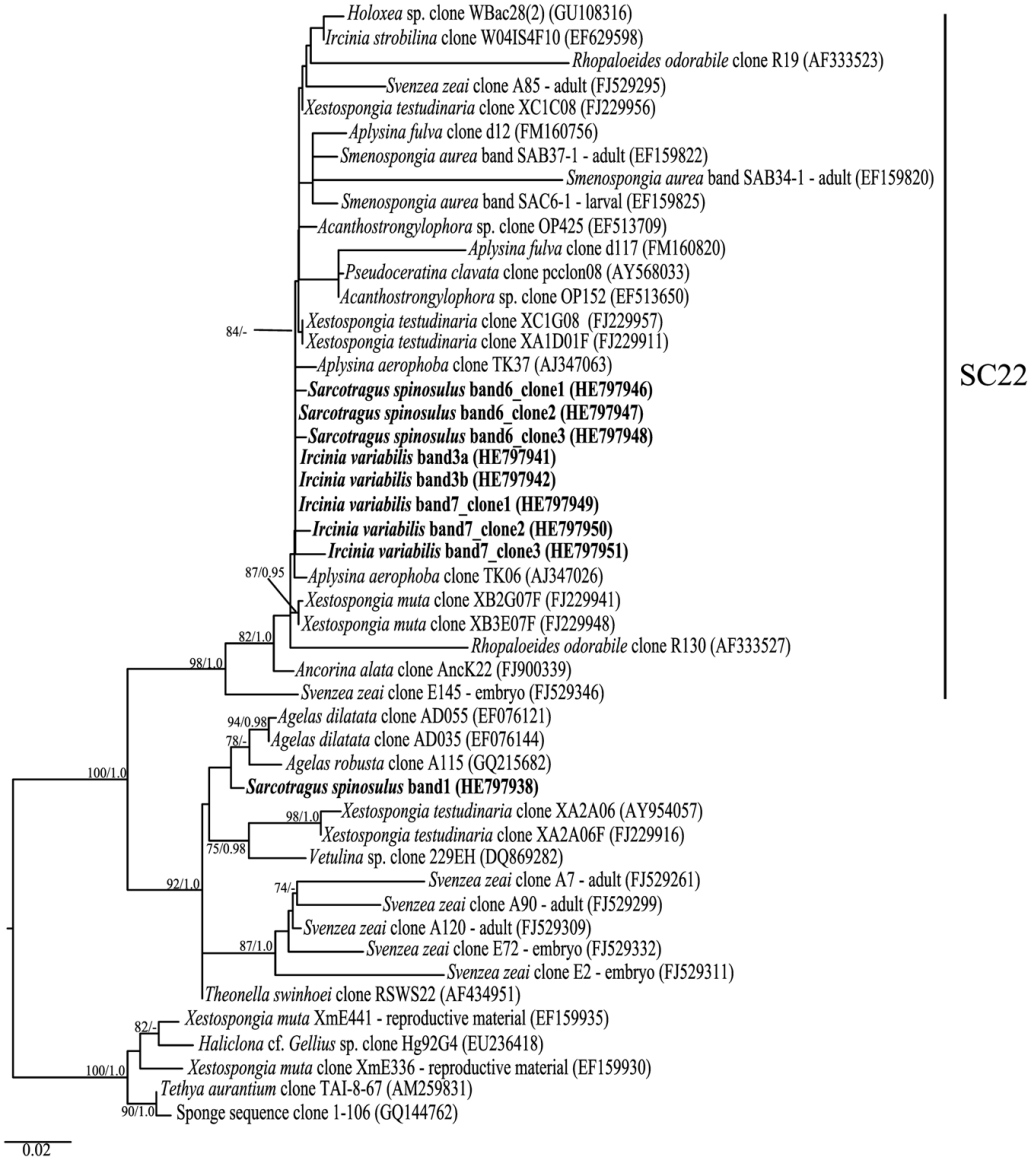
Phylogenetic inference of *Actinobacteria* 16S rRNA genes. The modified ARB database generated by Simister et al., [34] used long sequences (≥1200 bp) to infer the phylogeny and shorter sequences were added using the ARB parsimony interactive tool. Sequences from the sponge-specific cluster 22 (SC22) [34] along with sequences closely related to band 1 and outgroup sequences were selected for further phylogenetic analysis. The Maximum Likelihood tree (-ln likelihood: 4501,317092) is shown, with sequences retrieved in this study highlighted in bold. Numbers at tree nodes are bootstrap values and posterior probabilities calculated in Maximum Likelihood and MCMC Bayesian analyses, respectively, and values above 70/0.95 are shown.

**Figure 5:**
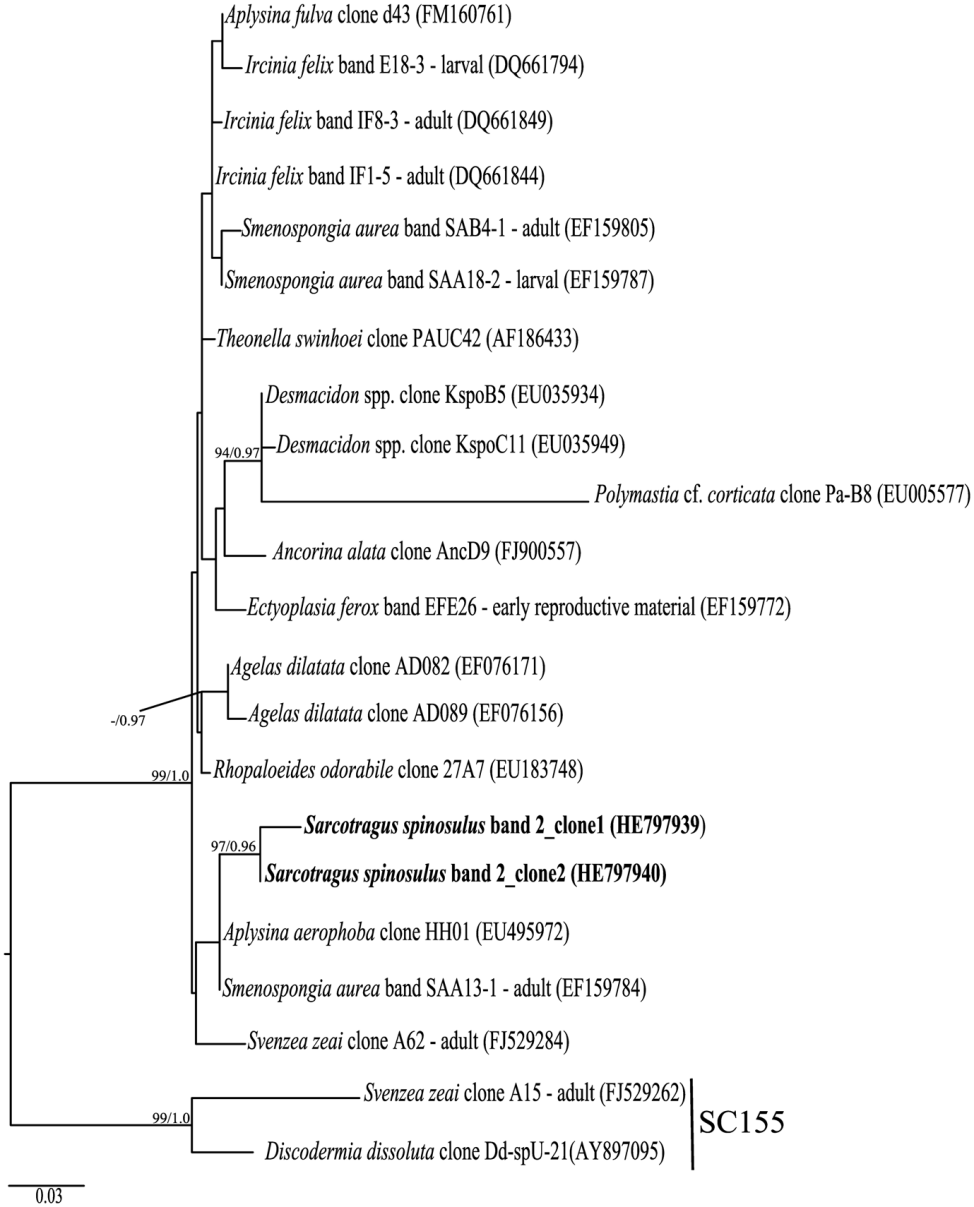
Phylogenetic inference of *Gammaproteobacteria* 16S rRNA genes. Tree construction procedure was as described for Figure 4, except that sequences closely related to band 2 were selected as well as sequences from SC155 [34], which were used as outgroup. The Maximum Likelihood tree is shown (-ln likelihood: 2696,593494).

**Figure 6:**
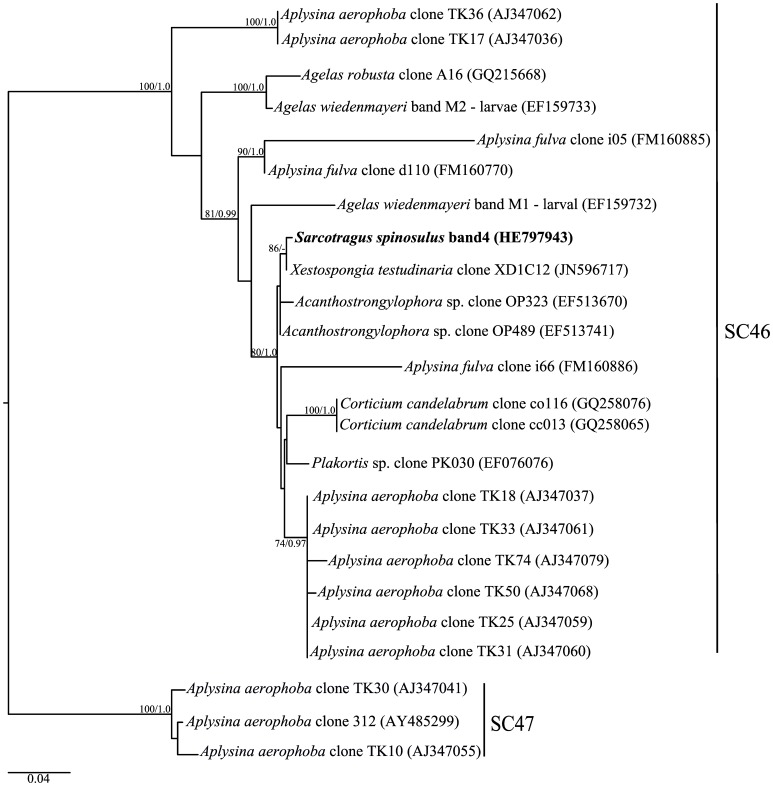
Phylogenetic inference of *Chloroflexi* 16S rRNA genes. Tree construction procedure was as described for Figure 4, except that sequences from SC46 were selected along with sequences from SC47, which were used as outgroup [34]. The Maximum Likelihood tree is shown (-ln likelihood: 3791,095).

Three dominant bands labelled 1, 2 and 4 (see arrows in Fig. 3a) were exclusively found in all replicates of *S. spinosulus*. Bands 1 and 4 were directly sequenced whereas band 2 was subjected to cloning and sequencing. From band 1, one sequence was retrieved and affiliated with the *Actinobacteria* order *Acidimicrobiales*. The phylogenetic analysis showed that this band affiliated with an uncultured and apparently diverse lineage containing sponge-derived bacterial sequences of worldwide origin (Fig. 4). Further, two clones were sequenced from band 2 and found to be highly alike, with 5 different nucleotides between them. They were assigned to the *Gammaproteobacteria* order *Chromatiales.* These sequences belonged to a well supported *Chromatiales* phylogenetic clade containing uncultured bacteria retrieved exclusively from marine sponges sampled in several geographical localities (Fig. 5). From band 4, one sequence was obtained and classified in the *Chloroflexi* phylum. Phylogenetic analysis revealed that this sequence belonged to a sponge-specific bacterial phylogenetic cluster as determined by Simister *et al*. [34] (Fig. 6). A dominant band labelled 3 (Fig. 3a) was found in all sponge specimens. Two identical sequences were recovered and assigned to the *Actinobacteria* order *Acidimicrobiales*. They also affiliated with an uncultured sponge-specific lineage previously suggested by Simister *et al*. [34] (Fig. 4). A dominant band labelled 5 (see arrow in Fig. 3a) was exclusively found in all seawater samples. This band possessed high discriminating power, as obviated by its centroid position in the CCA diagram. Two identical sequences were obtained for this band and assigned to the *Alphaproteobacteria* family *Rhodobacteraceae* (Table 1). They belonged to a supported, uncultured bacterial phylogenetic clade containing sequences retrieved solely from seawater (data not shown).

**Table 1:** Closest 16S rRNA gene relatives of seawater-derived and “cosmopolitan” PCR-DGGE bands.

Band id (Accession number)	RDP closest match^1^ (Accession number)	NCBI closest match^2^ (Percent similarity, accession number)
5a (HE797944)	Uncultured *Roseobacter* sp. (AY627365)	Uncultured *Rhodobacteraceae* bacterium clone GG101008Clone5 (99%, JN591908)
5b (HE797945)	Uncultured *Roseobacter* sp. (AY627365)	Uncultured *Rhodobacteraceae* bacterium clone GG101008Clone5 (99%, JN591908)
9a (HE797955)	Uncultured *Roseobacter* sp. (AY627365)	Uncultured *Rhodobacteraceae* bacterium clone GG101008Clone5 (99%, JN591908)
10a (HE797957)	Uncultured *Roseobacter* sp. (AY627365)	Uncultured *Rhodobacteraceae* bacterium clone GG101008Clone5 (100%, JN591908)
10b (HE797958)	Uncultured *Roseobacter* sp. (AY627365)	Uncultured *Rhodobacteraceae* bacterium clone GG101008Clone5 (100%, JN591908)
10c (HE797956)	Uncultured *Roseobacter* sp. (AY627365)	Uncultured *Rhodobacteraceae* bacterium clone GG101008Clone5 (100%, JN591908)

1 Closest 16S rRNA gene relative using the “sequence match” tool of the Ribosomal Database Project (RDP).

2 Closest 16S rRNA gene relatives as determined by the blast-n search in the National Center for Biotechnology Information (NCBI) database.

#### (ii) Actinobacteria PCR-DGGE bands

The dominant bands labelled 6 and 7 (Fig. 3C) were recovered from three specimens of *S. spinosulus* and from all *I. variabilis* specimens, respectively. These bands were subjected to cloning and sequencing. Three clones were obtained from each band, which contained 2 and 3 dissimilar nucleotides for bands 6 and 7, respectively. All sequences were assigned to the order *Acidimicrobiales*. Phylogenetic analysis revealed that these sequences fell into a sponge-specific bacterial phylogenetic clade [34] from which no cultured representative has so far been registered (Fig. 4).

#### (iii) Alphaproteobacteria PCR-DGGE bands

**Figure 7:**
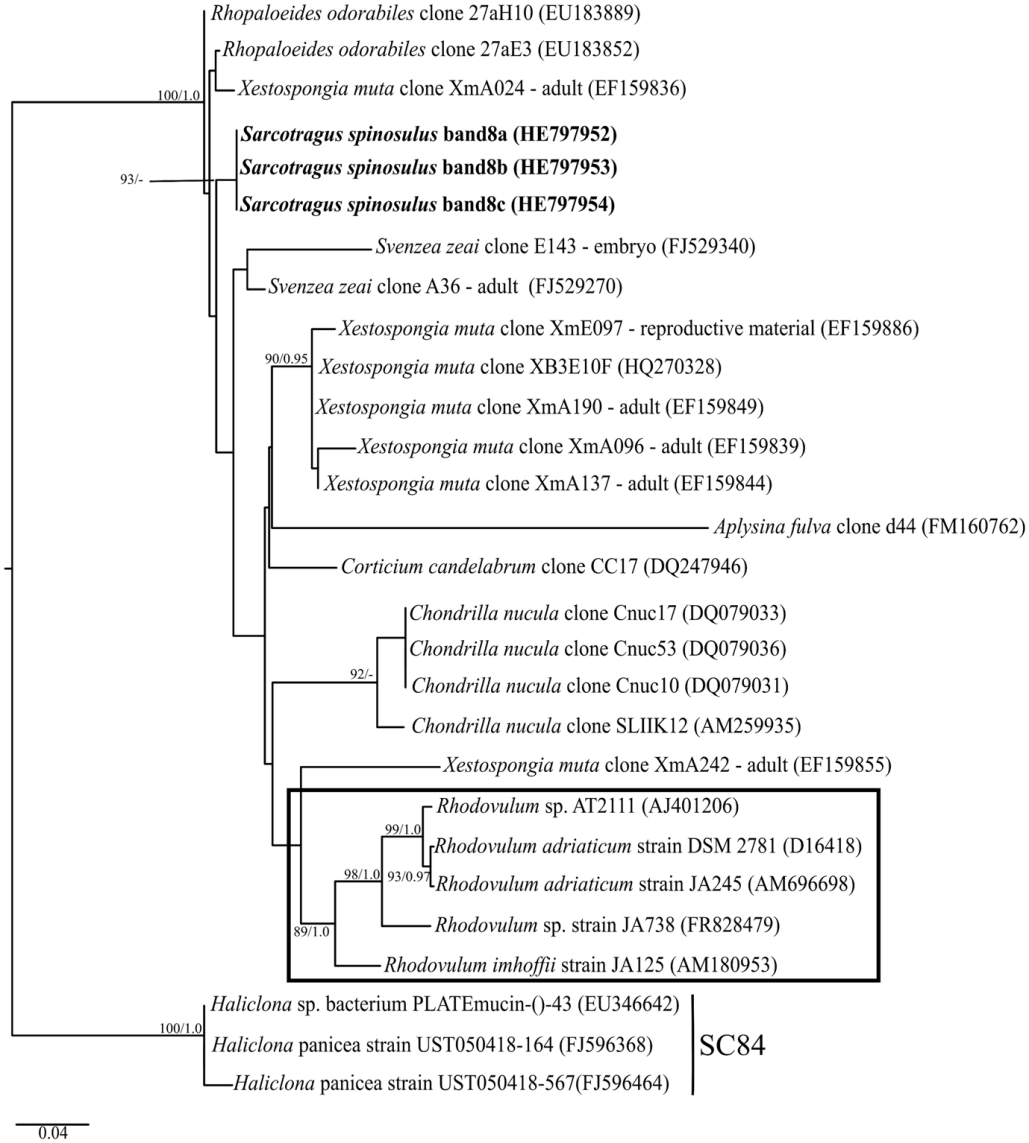
Phylogenetic inference of *Alphaproteobacteria* 16S rRNA genes. Tree construction procedure was as described for Figure 4, except that sequences close related to band 8 were selected along with sequences from SC84 [34], which were used as outgroup. The sequences shown in a box were isolated from different environments. The Maximum Likelihood tree is shown (-ln likelihood: 3253,686594).

The dominant band labelled 8 (Fig. 3e) appeared in all specimens of *S. spinosulus*. Three identical sequences were recovered and assigned to the order *Rhodobacterales*. Phylogenetic reconstruction revealed that these sequences affiliated with bacterial phylotypes retrieved almost solely from marine sponges distributed worldwide. No cultured representative isolated from marine sponges has been observed in this cluster (Fig. 7). One band found almost in all samples in addition to a dominant band found exclusively in seawater samples labelled, respectively, 9 and 10 (Fig. 3e) were subjected to sequencing. One and three sequences were obtained from bands 9 and 10, respectively. They all shared high similarity at the primary sequence level (up to 3 nucleotide differences detected), belonged to a phylogenetic cluster containing several uncultured bacterial phylotypes retrieved only from seawater, and affiliated with the family *Rhodobacteraceae* (Table 1).

## Discussion

This survey addresses bacterial abundance, diversity and specificity in the Atlanto-Mediterranean sponges *Sarcotragus spinosulus* and *Ircinia variabilis* (Demospongiae, Dictyoceratida, Irciniidae). These species are widely distributed along the southern Portuguese coast (http://www.marinespecies.org/porifera/). Both species were initially identified by traditional taxonomic methods. However, species within the Order Dictyoceratida to which the family *Irciniidae* belongs are, along with the Order Dendroceratida, known as ‘keratose’ sponges, which usually lack a suite of morphological features making their classification problematic [35], [36]. In recent years, molecular characterization of sponges by sequencing of standard genetic markers – known as DNA barcoding – is being used increasingly as a means to facilitate identification and to complement the description of new species [37]. Almost invariably, analyses involve the use of the subunit I of the cytochrome C oxidase gene (CO1) [37]–[39]. The genetic variation (p-distance) found in our host species’ CO1 gene, i.e. no intraspecific variation and a 4.7% genetic distance between *I.* *variabilis* and *S. spinosulus*, are within the range of values observed for other Irciniids using the same marker. In a barcoding study of Indo-Pacific Irciniids, Pöppe *et al.* [40] observed no intraspecific variation within any of the analysed species, low interspecific variation between congeners (0.2–2.7% in *Ircinia* spp. and 0.2–1.7% in *Psammocinia* spp.), and higher differentiation levels between members of the two genera (p-distances of 3.1–5.8%) [40]. In a second study comparing the bacterial symbionts in three species of *Ircinia* in the Mediterranean Sea, Erwin *et al*. [18] found no intraspecific variation within any of the studied species (nor between *I. variabilis*/*fasciculata*) and a p-distance of 0.6–1.8% between the different species. Not unexpectedly we found some genetic distance (p-distance 0–0.6%) between the sequences of our Atlantic specimens and those available on GenBank from Mediterranean specimens. This may indicate some level of genetic isolation and differentiation between conspecific populations occurring in these areas as previously observed in other sponge taxa (e.g. Xavier, *et al*. [41]). Overall, host phylogenetic inference can be a suitable and complementary tool in sponge microbiology studies - as shown in early [36], [42] and recent [18], [43] reports on host-symbiont co-evolutionary relationships. Its use seems especially well suited to the study of sponge hosts displaying smooth gradients of phylogenetic relatedness or unresolved taxonomies such as the members of the *Irciniidae* family and its relevance in such studies is likely to rise with the analysis of multiple phylogenetic markers in concatenation.

In the present survey, the abundance of culturable bacteria associated with *S. spinosulus* and *I. variabilis* was similar. It is well-known that many aspects affect bacterial cultivation and the use of standard culture media has so far allowed the assessment of only a minor fraction (e.g. from 0.1 to 1%) of bacteria associated with marine sponges [44]–[46]. This might sharply compromise the comparative assessment of bacterial abundance in sponges when solely using this technique. To circumvent the limitations inherent from cultivation, epifluorescence microscopy was applied to estimate abundance by enabling the count of all detectable nucleic-acid containing cells present in the samples. Based on the cell counts retrieved with this method, about 3 orders of magnitude higher than the registered CFU counts, *S. spinosulus* and *I. variabilis* can be regarded as HMA sponges, supporting previous observations obtained for tropical Irciniidae species such as *I. felix* and *I. strobilina* [12], [47], [48].

*Bacteria*, *Actinobacteria* and *Alphaproteobacteria* PCR-DGGE fingerprinting revealed a clear difference in bacterial diversity and community composition between sponge and seawater samples. This expected trend has been reported in several previous sponge microbiology surveys [1], [49], [50]. In agreement with our results, the bacterial PCR-DGGE profiles from *I. felix* collected at two sites in Key Largo, Florida, revealed distinct band patterns in comparison with seawater samples [12], whereas bacterial PCR-DGGE profiles of wild and captivated *I. strobilina* specimens were likewise distinct when compared with surrounding seawater and water used in sponge aquaculture, respectively [15]. In contrast with species of the genus *Ircinia*, knowledge of bacterial abundance and diversity in *Sarcotragus* specimens is virtually nonexistent. Here we showed *S. spinosulus*-specific profiles that strongly differed from those found in *I. variabilis*, with several species-specific PCR-DGGE bands detected and further identified (see below). *S. spinosulus* exhibited greater bacterial community diversity and richness, and homogeneity across individual specimens than the latter. Further, evidence for greater prokaryotic abundance in *S. spinosulus* was found. In a recent survey, Erwin *et al.* [18] could detect bacterial OTUs exclusive to the species *I. oros*, *I. fasciculata* or *I. variabilis* in the Mediterranean Sea. Taken together, these studies hint at a fundamental role of the host in shaping the structure and promoting diversity of symbiont communities within closely related sponge hosts. Interestingly, functional equivalence and evolutionary convergence of symbiont communities have been suggested as an evolutionary model applicable to the complex sponge microbiota, based on the share of core microbial functions between six phylogenetically distant sponge species with different symbiont community structures [51]. In this context, it is tempting to speculate that the less studied *Sarcotragus* also establishes close interactions with selected bacterial communities, which regardless their degree of distinctiveness might have intrinsic functions like those observed for *Ircinia* spp. [11], [15], [16]. Future studies addressing microbial functioning in sympatric and phylogenetically close hosts will certainly shed further light on our current understanding of symbiont evolution within sponges.

We successfully identified several sponge-specific bacterial populations by PCR-DGGE. Four dominant symbionts - two from *S. spinosulus*, one from *I. variabilis* and one found in both sponge species - were affiliated with an uncultured actinobacterial lineage within the order *Acidimicrobiales* [52]. Three of these (“bands” 3, 6 and 7 in Figs. 3 and 4) belonged to a cluster of sponge-specific sequences collected worldwide and called SC22 by Simister *et al*. [34], whereas the fourth grouped into a different and diverse cluster dominated by sponge-derived bacterial sequences (Fig. 4). Within cluster SC22, sequences were obtained from adult of *Svenzea zeai* and *Smenospongia aurea* along with their reproductive material, which suggests that vertical transmission of this particular phylotype is likely to occur [53], [54]. The same observation was made for the cluster formed by the fourth symbiont in the *Acidimicrobiales* group (“band 1” in Fig. 4) and related sequences, from which two sequences from adult *S. zeai* and one from its embryo were found [53]. These results indicate an intimate pattern of relationship between sponge-associated *Acidimicrobiales* and their hosts. The order *Acidimicrobiales* contains mesophilic and moderate thermophilic species and all members are obligatory acidophilic found in iron-, sulphur- or mineral-sulfide rich environments. Species within this order are capable of ferrous iron and sulphur oxidation and ferric iron reduction [55]–[58]. However, the physiological properties exhibited by cultivated *Acidimicrobiales* might not necessarily match those of marine sponge symbionts, as these usually share lower relatedness to cultured species at the 16S rRNA gene level, and therefore further research is needed to unveil the ecology and functioning of these symbionts in marine sponges.

A prevailing symbiont found exclusively in *S. spinosulus* was affiliated with uncultured *Gammaproteobacteria* within the order *Chromatiales* [59]. These sequences belonged to a cluster of sponge-specific sequences acquired worldwide (Fig. 5). Among them, adult sequences from *Ircinia felix*, *Smenospongia aurea* and *Svenzea zeai* were observed along with sequences from reproductive material of *I.* *felix*, *S. aurea* and *Ectyoplasia ferox* [11], [53], [54]. The order *Chromatiales* encompasses members of the purple sulphur bacteria that are capable of performing anoxygenic photosynthesis using hydrogen sulphide as electron donor [59]. Furthermore, many *Chromatiales* species have been shown to perform fixation of molecular nitrogen [59], [60]. These functions might be highly valuable for sponge survival, and the consistency with which members of this group are found in marine sponges at a global scale indeed suggests that *Chromatiales* species play an important role in their association with such hosts.

Another phylotype solely recovered from *S. spinosulus* was affiliated with an uncultured, sponge-specific lineage in the *Chloroflexi* phylum, named SC46 by Simister *et al*. [34] (Fig. 6). The *Chloroflexi* is regarded as one of the most abundant and diverse bacteria phyla associated with a wide variety of marine sponges, with many sponge-specific clusters identified [6], [34], [61]. So far, only one *Chloroflexi* species was isolated from the sponge *Geodia* spp., which also clustered with sequences exclusively obtained from marine sponges [62]. In shallow waters, members of *Chloroflexi* are able to fix atmospheric carbon through photosynthesis, and thus these bacteria could provide carbonaceous compounds to the sponge host [62]. Recently, a *Chloroflexi* bacterium was pointed as the likely producer of a novel non-ribosomal peptide synthase [63]. Thus, *Chloroflexi* strains might play important roles in sponge nutrition and defence.

Using a taxon-specific fingerprinting approach to the *Alphaproteobacteria,* a dominant symbiont exclusive to *S. spinosuls* was uncovered (“band 8” in Fig. 3) and found to be closely related to an uncultured alphaproteobacterium within the family *Rhodobacterales* [64]. Sequences representing this symbiont formed a concise cluster with sequences retrieved from marine sponges in several geographical backgrounds in addition to cultured representatives obtained from different environments such as microbial mats, seawater, soil from saltpan, water and marine aquaculture pond [65], [66] (Fig. 7). This clade contained sequences obtained from adults of *Xestospongia muta* and *Svenzea zeai* along with their reproductive material [53], [54]. This symbiont is closely related to *Rhodovulum* species, in which many type strains have been mostly retrieved from marine habitats. This genus contains species that undertake diverse metabolic pathways such as photoautotrophic, photoheterotrophic and chemotrophic and occur mainly in marine and hypersaline environments under oxic, microoxic and anoxic conditions [67]. The metabolic versatility of *Rhodovulum* species indicate that they are able to use the waste generated by sponges. For instance, ammonia, which is a toxic metabolic waste product that could accumulate within the sponge body, especially during low pumping activity, might be used as nitrogen source for *Rhodovulum* species [1], [67], [68]. In addition, some strains of *Rhodovulum* could be involved in nitrogen and sulphur cycling, once they are capable to use dinitrogen, sulphur, sulphite, sulphate and thiosulfate [67]. Vertical transmission has also been documented for members of this genus in marine sponges, [54] suggesting *Rhodovulum* as a likely, relevant constituent of the sponge-associated microbiome.

The present study provides first insights into the bacterial abundance and diversity in Atlantic *S. spinosulus* and *I. variabilis*. In spite of their sympatric occurrence, the inspected species hosted bacterial communities that differ from each other and from those found in seawater. Interestingly, all bands excised from PCR-DGGE profiles that were exclusive to sponge samples affiliated with previously identified sponge-specific sequence clusters [34] or with potentially novel sponge-specific clusters found in the present survey. Thus, the approach used here enabled not only straightforward assessment of overall trends in bacterial community structures, but also direct identification of symbionts of putative relevance in association with their hosts, given their dominance and consistent patterns of occurrence in the analysed specimens, and their presumed sponge-specific life histories as inferred by 16S rRNA gene phylogenies. Notably, bacterial phylotypes regarded as “*S. spinosulus*-specific” or “*I. variabilis*-specific” in this study shared high degrees of resemblance with sponge-derived sequences from other biogeographical settings and/or more distantly related sponge hosts. This picture, in which bacterial signatures not shared by co-occurring and taxonomically close sponge species are found in disparate sponge hosts and localities, most likely derives from factors of the host and of the environment – including vertical transmission *vs.* environmental acquisition of symbionts, specific habitat preferences and life stages of the host - that cooperatively shape the structure of the sponge-associated microbiome [1], [18]. As a result, complex communities of specific composition at the host species or even specimen level [9], [28] with concomitant sharing, across sponge species, of generalist symbionts displaying broad host range and/or widespread occurrence [43], [69] have often been reported for marine sponges. Here, the distinct communities observed in *S. spinosulus* and *I. variabilis* within the same habitat, along with the detection of symbionts showing broad host and geographical ranges as inferred by 16S rRNA gene phylogenies, hints at a pivotal role for the host in shaping the structure of its own microbiota while revealing versatile and widespread bacterial phylotypes with apparently intimate sponge-associated life histories. The high abundance and species-specific character of these assemblages suggest in-faunal microbial communities as overriding drivers of functioning and of genetic and metabolic diversities in coastal ecosystems.

## Materials and Methods

### 

#### Sponge and seawater sampling

Four specimens of *Sarcotragus spinosulus* and *Ircinia variabilis* (Demospongiae, Dictyoceratida, Irciniidae) were collected by scuba diving at depths around 15 m at Galé Alta, Armação de Pêra (37° 04′ 09.6′′N and 8° 19′ 52.1′′W) in the coast of the Algarve, Portugal, in June 2010. Measurements of temperature, oxygen and salinity during the sampling procedure were 14.6°C, 5.95 mg L^−1^ and 35.11 part per million (ppm), respectively. *In situ* pictures of the specimens were taken to aid laboratory identification (Fig. S1). The individual samples were placed, *in situ*, separately in plastic bags (type ziploc®) containing natural seawater, transferred into cooling boxes, brought to the laboratory within few hours and processed upon arrival. Four samples of seawater (1L each) from the vicinity of the sponges (i.e. 1 m above the specimens) were also collected as above. Prior to sample processing, the sponge specimens were rinsed with sterile Artificial Seawater (ASW) [70] to remove loosely associated organisms. Voucher samples were preserved in 90% ethanol for taxonomic identification and deposited in the Biology Department´s zoological collection of the University of the Azores (DBUA.Por). Because sampling did not involve endangered or protected species and did not occur within privately owned or protected areas, no specific permits were required for the described field studies.

#### Sponge identification

Specimens were identified from the analysis of general external morphological characters and internal skeletal features, i.e. thickness, degree of fasciculation and presence of foreign debris within the spongin fibres and structure of the collagenous filaments. Genera within the family Irciniidae are distinguished by the presence of a cortical armour of sand (exclusive to *Psammocinia*), and presence (in *Ircinia*) versus absence (in *Sarcotragus*) of foreign debris within the primary fibres [35].

Phylogenetic inference of sponge specimens (commonly referred to as “sponge DNA barcoding”) was used to aid species identification by molecular means. PCR amplifications were carried out on sponge total community DNA (see below) targeting the subunit I of the cytochrome oxidase gene with the primers dgLCO1490 and dgHCO2189 [71]. This fragment (*c.* 640 bp) encompasses the standard “barcoding” partition [72]. The reaction mixture (25 µL) contained 1.5 µL of template DNA (∼20 ng), 1X reaction buffer (Bioline, London, UK), 0.16 mM deoxynucleoside triphosphates (dNTPs), 4.0 mM MgCl_2,_ 0.64 mg mL^−1^ of bovine serum albumin (BSA), 0.24 µM of primers and 0.625U of Bio*Taq*™ DNA polymerase (Bioline, London, UK). After initial denaturation at 95°C for 3 min., 36 cycles of 45 sec at 94°C, 60 sec at 51°C and 90 sec at 72°C were carried out. A final extension of 10 min at 72°C was used to finish the reaction. All PCR amplifications were carried out in a MyCycle thermal cycler (Bio-Rad, Hercules, CA, USA). Amplicons were checked after electrophoresis on 1% agarose gel under UV light. PCR products with right size were cleaned with Sephadex G50 (GE Healthcare Bio-Science AB, Uppsala, Sweden) columns, quantified with Image Lab™ Software (Bio-Rad, Hercules, CA, USA), and subjected to sequencing with the chain termination method in an Applied Biosystems 3130 genetic analyser using the forward primer. Closest relatives were searched using the megablast and blastn algorithms of the National Center for Biotechnology Information (NCBI) [73]. Closely related sequences from the NCBI and the Sponge Barcoding Project (www.spongebarcoding.org) databases were used to retrieve representative CO1 sequences for phylogenetic inference (see below).

#### Plate counting of heterotrophic bacteria

Per sponge specimen, 2.5 g of fresh internal body was cut and transferred to a 50 mL screw cap polypropylene tube containing 25 mL of Calcium/Magnesium Free Artificial Seawater (CMFASW) [74]. The sponge samples were ground with sterile mortar and pestle. The resulting suspensions were collected and allowed to decant for 5 min. Serial 10-fold dilutions were then prepared with sterile ASW and plated in triplicate onto Marine broth (Carl Roth GmbH+Co, Germany) plus 1.5% agar. The plates were incubated at room temperature (∼25°C) and Colony Forming Unit (CFU) counting was performed after 3, 5 and 7 days of incubation. Log-transformed CFU values fitted the normal distribution and were compared by One Way Analysis of Variance (ANOVA) using PASW Statistics 18 (SPSS Inc., Chicago, USA).

#### Epifluorescence microscopy

A cultivation-independent analysis of prokaryotic abundance based on epifluorescence microscopy was performed in this study. For the sponge samples, the suspensions prepared in the abovementioned procedure were first centrifuged at 500 g for 2 min to remove sponge cells and debris. Aliquots (100 µL) of the resulting supernatants were individually fixed in 2.5% glutaraldehyde and the volume was completed to 10 mL with sterile ASW. Seawater samples (9.2 mL) were also fixed in 2.5% glutaraldehyde. From the fixed material, 100 µL and 10 mL from sponge and seawater samples, respectively, were filtered through 0.2-µm-pore-size isopore™ black membrane filters (Millipore, Bellerica, MA, USA). The filter was stained with the DNA-binding fluorochrome acridine orange, mounted on glass slides and analysed with an inverted research system microscope IX81 (Olympus Europa GmbH, Hamburg, Germany) where 25 photos per specimen were taken at random. Cells with a well-defined edge, usually ranging from 0.2 to 1 µm in diameter when coccoid, or reaching up to 5 µm in length when rod-shaped, were counted and served as proxy for prokaryotic cell abundance in the samples. Larger objects (>5 µm) that could eventually account for eukaryotic organisms were not considered. Total prokaryotic numbers were log-transformed and analysed by One Way ANOVA using PASW Statistics 18 (SPSS Inc., Chicago, USA).

#### Total community DNA extraction

Genomic DNA of about 0.25 g of internal sponge body was extracted using UltraClean® Soil DNA isolation kit (Mo Bio, Carlsbad, CA, USA) according to the manufacturer’s protocol. Based on preliminary PCR-DGGE assessments, this method led to a more reproducible depiction of bacterial community structures in the sponges when compared with a method that employs a cell-separation treatment prior to DNA extraction (Hardoim et al., unpublished results), and was therefore chosen for the purpose of this study. Seawater samples (500 mL) were filtered through 0.2-µm-pore-size nitrocellulose filters (Millipore, Billerica, MA, USA) using a vacuum pump. The filters were cut into small pieces and directly used for DNA extraction as explained above.

#### Bacterial PCR for DGGE analysis

A nested PCR-denaturing gradient gel electrophoresis (PCR-DGGE) approach was chosen - based on its higher detection sensitivity and reproducibility when compared with a one-step amplification protocol in preliminary assays (data not shown) - to assess the total bacterial communities in all samples. Nearly full-length 16S rRNA gene fragments were amplified with the primer pair F27 and R1492 [75]. The reaction mixture (25 µL) was prepared with 1 µL of template DNA (∼20 ng), 1X Stoffel buffer (Applied Biosystems, Foster, CA), 0.2 mM dNTPs, 3.75 mM MgCl_2,_ 0.1 mg mL^−1^ of BSA, 2% (vol/vol) dimethyl sulfoxide (DMSO), 0.2 µM of each primer, and 1.25U of *Taq* DNA polymerase (Applied Biosystems, Foster, CA). After initial denaturation at 94°C for 5 min, 30 cycles of 1 min at 94°C, 1 min at 56°C and 2 min at 72°C were performed, followed by a final extension for 10 min at 72°C. The amplicons (1.5 µL) were used as template in a subsequent PCR for DGGE analysis (20 cycles) using the primer pair F984-GC and R1378 [76]. The PCR mixture and thermal cycling followed the protocol by Costa *et al*. [77], with half the quantity of *Taq* DNA polymerase (1.25 U) per reaction.

### PCR of Specific Bacterial Groups for DGGE Analyses

#### Actinobacteria 16S rRNA gene fragments

The first amplification of the nested PCR was carried out with the primers F243 [76] and R1494 [75] to generate *Actinobacteria*-specific amplicons. The reaction mixture and PCR conditions were carried out as described by Hardoim *et al*. [6], except for the concentration of *Taq* DNA polymerase (1.25U), number of cycles (25 cycles) and extension period (1 min) in the present study. The amplicons (2 µL) were used in a second PCR for DGGE analysis using the primers F984-GC and R1378 [76] as described previously for total bacteria, except for the number of cycles (30 cycles).

#### Alphaproteobacteria 16S rRNA gene fragments

The first reaction mixture of the nested PCR was prepared as described by Gomes *et al*. [78], except that in the present study the primer concentration and *Taq* DNA polymerase were 0.2 µM and 1.25 U, respectively, and that BSA was not used in the group-specific PCR. After initial denaturation at 94°C for 7 min, 30 cycles of 1 min at 94°C, 1 min at 56°C and 1 min at 72°C were carried out. The reaction was finished with an extension of 10 min at 72°C. Amplicons from the first reaction (2 µL) were used in a subsequent PCR for DGGE analysis as described previously, except for the number of cycles (25 cycles).

#### PCR-DGGE profiling

DGGE assays were carried out in a PhorU-2 gradient system (Ingeny International, Goes, The Netherlands). The 16S rRNA gene amplicons generated as explained above were applied in even concentrations onto polyacrylamide gels containing a 46.5 to 65% gradient of denaturants (100% denaturants defined as 7 M urea and 40% formamide) and a 6.2 to 9% gradient of acrylamide. Mixtures of PCR products of ten bacterial strains isolated from *Sarcotragus* sp. and *Ircinia* sp. (*Staphylococcus* sp.; *Ruegeria* sp.; *Pseudomonas* sp.; *Leisingera* sp.; *Corynebacterium* sp.*; Micrococcus* sp.*; Streptomyces* sp. and *Pontibacter* sp.) were loaded at the edge of the gels as markers. Electrophoresis was performed in a 1X Tris-acetate-EDTA buffer (pH 7.8) at 58°C and 140V for 16 h. The gels were silver stained [76] and air dried, after which digital images were obtained by scanning.

#### Analysis of PCR-DGGE profiles

The software GelCompar II 5.1 (Applied Maths, Kortrijk, Belgium) was used to analyse the PCR-DGGE profiles as recommended by Rademaker and de Bruijn, [79]. Briefly, pairwise Pearson correlation coefficients (r) were calculated as a measurement of the similarity between the community profiles. Cluster analysis was carried out with the unweighted pair group method with mathematical averages (UPGMA) using the similarity matrix generated with the calculated Pearson coefficients. In addition to cluster analysis, constrained (i.e. canonical) ordination was performed with the Canoco for Windows 4.5 software package (Microcomputer Power. Ithaca, NY) using a “sample×species” datasheet as input, in which the “species” data represent the presence and relative abundance of PCR-DGGE bands in each fingerprint, as described in detail by Costa *et al*. [80]. This was used to infer whether sponge species and seawater significantly contributed to the observed variability in the PCR-DGGE profiles (see [6], [80]). The Shannon measure of diversity (H’), determined as *H*’ = –∑ pi.logpi where pi represents the relative abundance of the i^th^ category (i.e. PCR-DGGE band) within the sample (i.e. PCR-DGGE fingerprint) was applied to estimate the diversity of each PCR-DGGE fingerprint generated in this study. The evenness (J’ = H’/Hmax) of PCR-DGGE fingerprints was calculated based on the diversity indices obtained. Measures of richness (i.e. number of PCR-DGGE bands), diversity and evenness of PCR-DGGE fingerprints were compared by One Way ANOVA using PASW Statistics 18 (SPSS Inc., Chicago, USA).

#### Identification of dominant bands in PCR-DGGE profiles

Sponge-associated and seawater exclusive bands were visually determined based on their occurrence across replicates (i.e. only bands detected in at least 3 of 4 replicates were sequenced). Further, their discriminating power was assessed, and only bands displaying high variance in relative abundance as a response to the sample classes “*I. variabilis*”, “*S. spinosulus*” and “Seawater” were selected. Discriminating bands were revealed by the species fit range function in the Canoco for windows 4.5 software, where only those bands displaying 50% fit range or more were considered for sequencing purposes. Discriminating bands were excised from DG-gels and re-amplified for PCR-DGGE analysis using the method of Costa *et al*. [80]. The resulting amplicons were loaded onto DGGE with the original community DNA samples to verify their electrophoretic mobility. Excised bands that displayed the right melting behaviour when compared with the original band in the community profiles were used as templates in another PCR amplification, in which the forward primer F984 used had no GC clamp. PCR-DGGE reaction mixtures and thermal cycles were carried out as described above. The amplicons were then purified in Sephadex G50 columns, quantified with Image Lab™ Software, and subjected to sequencing as above mentioned. For some excised bands, no pure amplicon was recovered and thus a cloning procedure was undertaken using the pGEM-T Vector System II Kit (Promega, Madison, WI) as described elsewhere [6], [77], [80]. Clones that showed the same electrophoretic mobility when compared to their original band were selected for sequencing as explained above. All sequences retrieved in this study were submitted to the EMBL Nucleotide Sequence Database under accession numbers HE797930 to HE797937 for sponge CO1 sequences and HE797938 to HE797958 for PCR-DGGE bands representing bacterial 16S rRNA genes.

#### Phylogenetic analyses

Sequences generated from sponge CO1 amplification and excised bacterial bands were quality-inspected and edited with the Sequence Scanner software V.1 (Applied Biosystems). For bacterial DGGE bands, taxonomic assignment of sequences was performed with the seqmatch and classifier tools of the Ribosomal Database Project II (http://rdp.cme.msu.edu) at 80% confidence threshold. Closest phylogenetic relatives were searched with the blast-n algorithm of NCBI. PCR-DGGE band sequences and their closest phylogenetic relatives were aligned using the SINA web aligner [81]. Aligned sequences were then imported into a modified SILVA 16S rRNA gene database version 102, which included all sponge-derived 16S rRNA gene sequences available in early 2010 [34], using the parsimony tool as implemented in the ARB software [82]. The sponge database generated by Simister *et al*. [34] contained phylogenetic inferences performed with long sequences (≥1200bp) using the program RAxML for all sponge-associated bacterial phyla, from which sponge-specific clusters were assigned [34] according to the criteria described by Hentschel *et al*. [69]. Alignments were manually checked and corrected when necessary using the ARB alignment window. The sequences generated in this study were added to maximum likelihood trees inferred by Simister *et al*. [34] through the parsimony interactive tool available in ARB using 50% conservation filters for each of the corresponding bacterial phyla, and their affiliation with sponge-specific phylogenetic clusters was then ascertained. From the resulting trees, relevant sequences were selected for further phylogenetic analyses (see below). The CO1 gene sequences from each investigated specimen were aligned against selected sponge barcoding sequences using Clustal X in the MEGA5 software [83]. Phylogenetic inferences of bacteria and sponge sequences were performed as described by Hardoim *et al*. [84]. Briefly, an appropriate evolutionary model for all phylogenetic trees was determined using ModelGenerator version 85 [85] and found to be the general-time reversible model (GTR, [86] with a discrete gamma-distribution of among-site rate variation (Γ_4_) and a proportion of invariant sites (I), except for CO1 inference, in which invariant sites did not fit. Maximum likelihood and Bayesian MCMC analyses were conducted using RAxML (vers. 7.0.4-MPI) and MrBayes (vers. 3.2.1), respectively [87]–[89].

## Supporting Information

Figure S1 **Sponge species.** *In situ* pictures of *S. spinosulus* (A) and *I. variabilis* (B).
(TIF)

Figure S2 **Cluster analysis.** Cluster analysis of PCR-DGGE fingerprints obtained for *Bacteria* (A), *Actinobacteria* (B) and *Alphaproteobacteria* (C). *S. spinosulus*: Alg10/08, Alg10/09, Alg10/10 and Alg10/11; *I. variabilis*: Alg10/12, Alg10/13, Alg10/14 and Alg10/15 and Seawater: SW07, SW22, SW23 and SW24.
(TIF)

Table S1 **PCR-DGGE band richness, diversity and evenness.**
(DOCX)
