# Supplementary figures and images for: Phylogenetically and Spatially Close Marine Sponges Harbour Divergent Bacterial Communities
Source: PLoS One. 2012 Dec 27;7(12):e53029. doi: 10.1371/journal.pone.0053029 (PMC3531450; doi:10.1371/journal.pone.0053029)

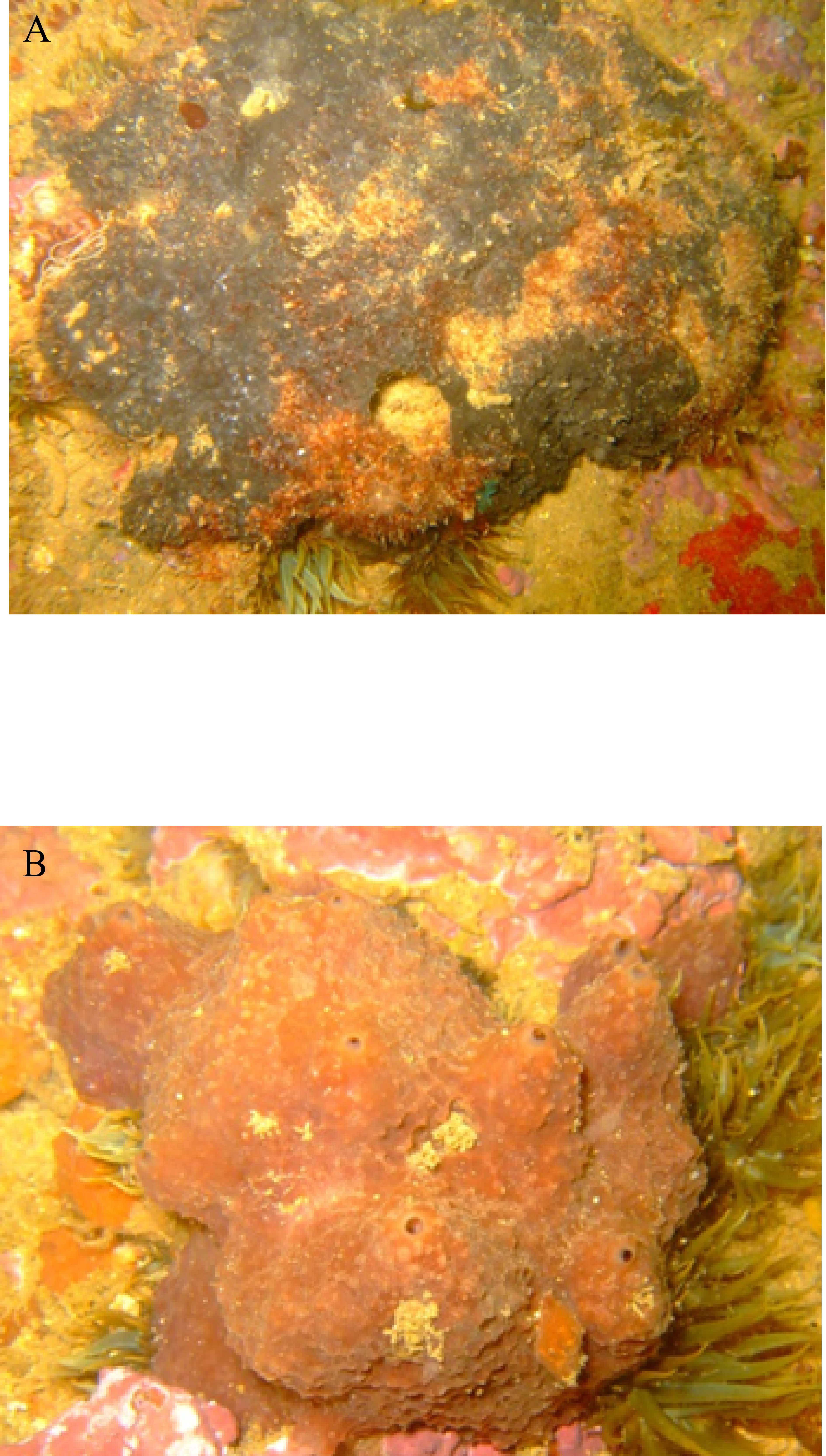

Supplement: Figure S1 — Sponge species. In situ pictures of S. spinosulus (A) and I. variabilis (B). (TIF) [file pone.0053029.s001.tif]

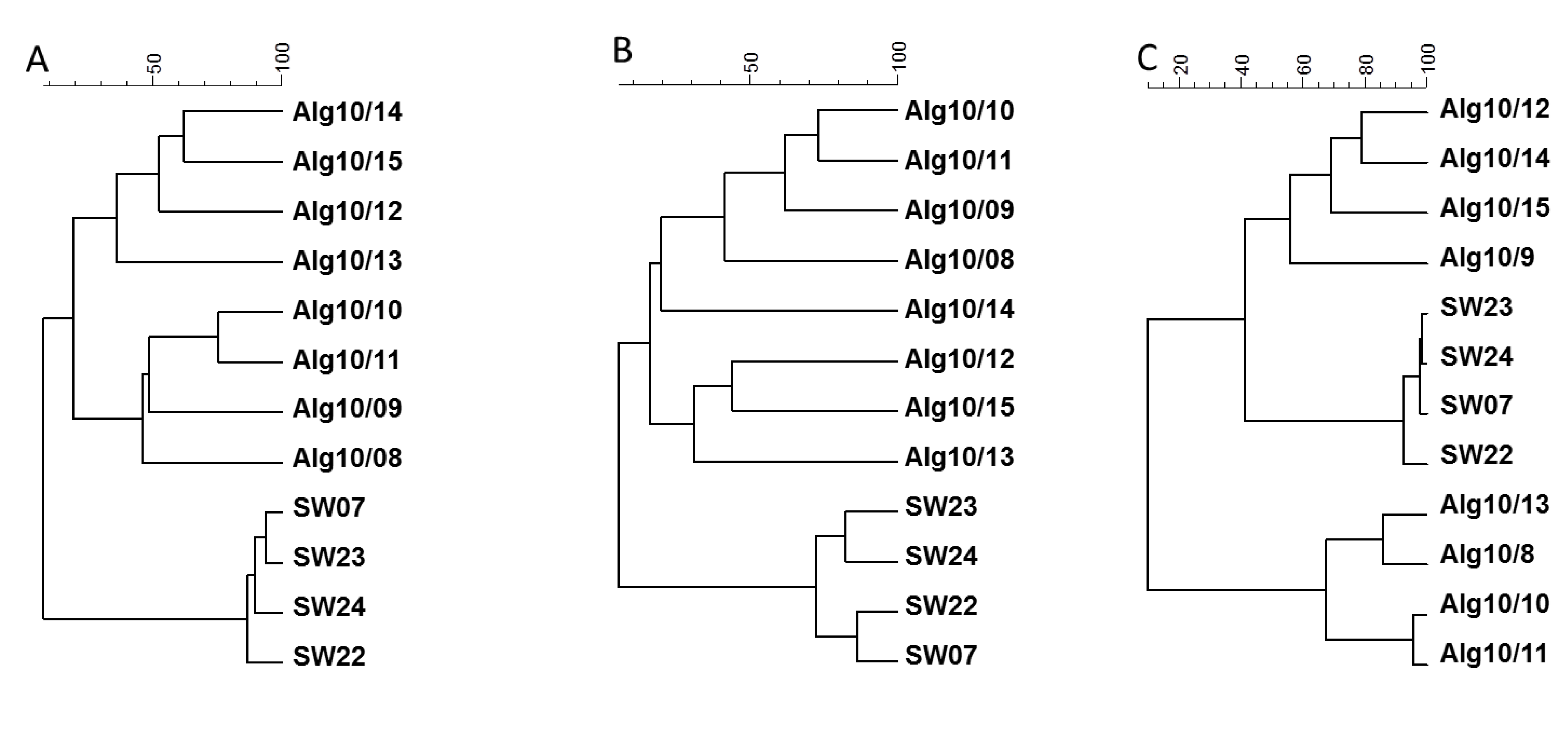

Supplement: Figure S2 — Cluster analysis. Cluster analysis of PCR-DGGE fingerprints obtained for Bacteria (A), Actinobacteria (B) and Alphaproteobacteria (C). S. spinosulus: Alg10/08, Alg10/09, Alg10/10 and Alg10/11; I. variabilis: Alg10/12, Alg10/13, Alg10/14 and Alg10/15 and Seawater: SW07, SW22, SW23 and SW24. (TIF) [file pone.0053029.s002.tif]
