# Supplementary material for: Phylogenetically and Spatially Close Marine Sponges Harbour Divergent Bacterial Communities
Source: PLoS One. 2012 Dec 27;7(12):e53029. doi: 10.1371/journal.pone.0053029 (PMC3531450; doi:10.1371/journal.pone.0053029)
Supplement: Table S1 — PCR-DGGE band richness, diversity and evenness. (DOCX) [file pone.0053029.s003.docx]

| ***Bacteria PCR-DGGE*** | **Richness** | **Diversity** | **Evenness** |
| --- | --- | --- | --- |
| *S. spinosulus* | 28.25 ± 2.93 ^a^ | 3.03 ± 0.15 ^a^ | 0.91 ± 0.03 ^a^ |
| *I. variabilis* | 15.25 ± 4.52 ^b^ | 2.15 ± 0.52 ^a^ | 0.79 ± 0.12 ^a^ |
| Seawater | 36 ± 0.58 ^a^ | 3.18 ± 0.09 ^a^ | 0.88 ± 0.02 ^a^ |
| ***Actinobacteria PCR-DGGE*** | **Richness** | **Diversity** | **Evenness** |
| *S. spinosulus* | 10.75 ± 0.94 ^a^ | 2.10 ± 0.05 ^a^ | 0.89 ± 0.03 ^a^ |
| *I. variabilis* | 5.00 ± 1.29 ^b^ | 1.31 ± 0.27 ^b,c^ | 0.88 ± 0.03 ^a^ |
| Seawater | 12.25 ± 1.03 ^a^ | 1.51 ± 0.44 ^a,c^ | 0.79 ± 0.02 ^a^ |
| ***Alphaproteobacteria PCR-DGGE*** | **Richness** | **Diversity** | **Evenness** |
| *S. spinosulus* | 11.50 ± 1.44 ^a^ | 1.66 ± 0.07 ^a^ | 0.69 ± 0.03 ^a^ |
| *I. variabilis* | 4.00 ± 0.71 ^b^ | 1.12 ± 0.18 ^b^ | 0.82 ± 0.04 ^b^ |
| Seawater | 10.00 ± 0.71 ^a^ | 2.19 ± 0.08 ^c^ | 0.95 ± 0.01 ^c^ |

^1^ Values are expressed as means ± standard error of the mean. Within each group of values, those represented by different letters are significantly different as determined by One Way ANOVA (*p*<0.05).
